# Retraction Note: Mechanisms of nanosized titanium dioxide-induced testicular oxidative stress and apoptosis in male mice

**DOI:** 10.1186/s12989-015-0098-0

**Published:** 2015-07-14

**Authors:** Xiaoyang Zhao, Lei Sheng, Ling Wang, Jie Hong, Xiaohong Yu, Xuezi Sang, Qingqing Sun, Yuguan Ze, Fashui Hong

**Affiliations:** Medical College of Soochow University, Suzhou, 215123 China; Library of Soochow University, Suzhou, 215123 China; Jiangsu Province Key Laboratory of Stem Cell Research, Soochow University, Suzhou, 215007 China; Cultivation base of State Key Laboratory of Stem Cell and Biomaterials built together by Ministry of Science and Technology and Jiangsu Province, Suzhou, 215007 China

## Retraction Note

This article [1] has been retracted by the Editor. A committee at Soochow University has investigated this case and supports the decision to retract the article. Incorrect statistical methods were used to calculate mean and S.D. values and additional errors were made in determining 8-OHdG concentrations. It has come to light that Figure 1 and Figure 2 were published in a previous article [2]. The committee also found that some of the original data were missing. We apologize to the readership of *Particle and Fibre Toxicology*.
